# Lenalidomide-Associated Hemophagocytic Lymphohistiocytosis With Plasma Cell Phagocytosis

**DOI:** 10.7759/cureus.14409

**Published:** 2021-04-10

**Authors:** Elliot Runge, Chung-ting J Kou, Matthew Rendo, David Lynch, Joshua Fenderson

**Affiliations:** 1 Internal Medicine, Brooke Army Medical Center, San Antonio, USA; 2 Hematology and Oncology, Brooke Army Medical Center, San Antonio, USA; 3 Pathology and Laboratory Medicine, Brooke Army Medical Center, San Antonio, USA

**Keywords:** hemophagocytic lymphohistiocytosis (hlh), diagnosis of multiple myeloma, secondary hlh, lenalidomide

## Abstract

Hemophagocytic lymphohistiocytosis (HLH) is a severe systemic inflammatory syndrome that is often fatal. In the adult population, it is believed to develop secondary to immune dysregulation due to rheumatologic, infectious, malignant, and recently, immunomodulatory drugs. It’s co-occurrence with phagocytosis by non-macrophage cells has not been previously well defined. We present a case of lenalidomide-associated HLH with concurrent plasma cell hemophagocytosis in a patient with controlled multiple myeloma (MM).

## Introduction

Hemophagocytic lymphohistiocytosis (HLH) is an aggressive immunologic hyper activation syndrome resulting in often life-threatening inflammation, hypercytokinemia, hemophagocytosis, and multi-organ failure [1,2]. It is defined by macrophage phagocytosis of erythrocytes, leukocytes, platelets, and cellular precursors in the bone marrow and other tissue. HLH can be categorized as either a primary or a secondary syndrome [1,2]. Primary hemophagocytosis usually presents in early childhood and results in cytotoxic impairment in natural killer (NK) cells [1,2]. Secondary HLH is associated with predisposing conditions, such as rheumatologic, malignant, or infectious etiologies. The diagnosis of HLH requires meeting pre-established criteria, which also includes molecular testing [3,4]. Here we present a case of lenalidomide-associated HLH in a patient with controlled multiple myeloma (MM) with concurrent plasma cell hemophagoctosis.

## Case presentation

A 70-year-old female presents with a history of MM. She previously received six cycles of bortezomib, cyclophosphamide, and dexamethasone (RVD) followed by melphalan myeloablative conditioning with an autologous stem cell transplant (ASCT). She had been on a stable maintenance dose of lenalidomide for three years at the time of presentation for a three-day history of falls and confusion. Of note, the patient had consistently normal cell lines at baseline one to two months prior to presentation.

**Figure 1:**
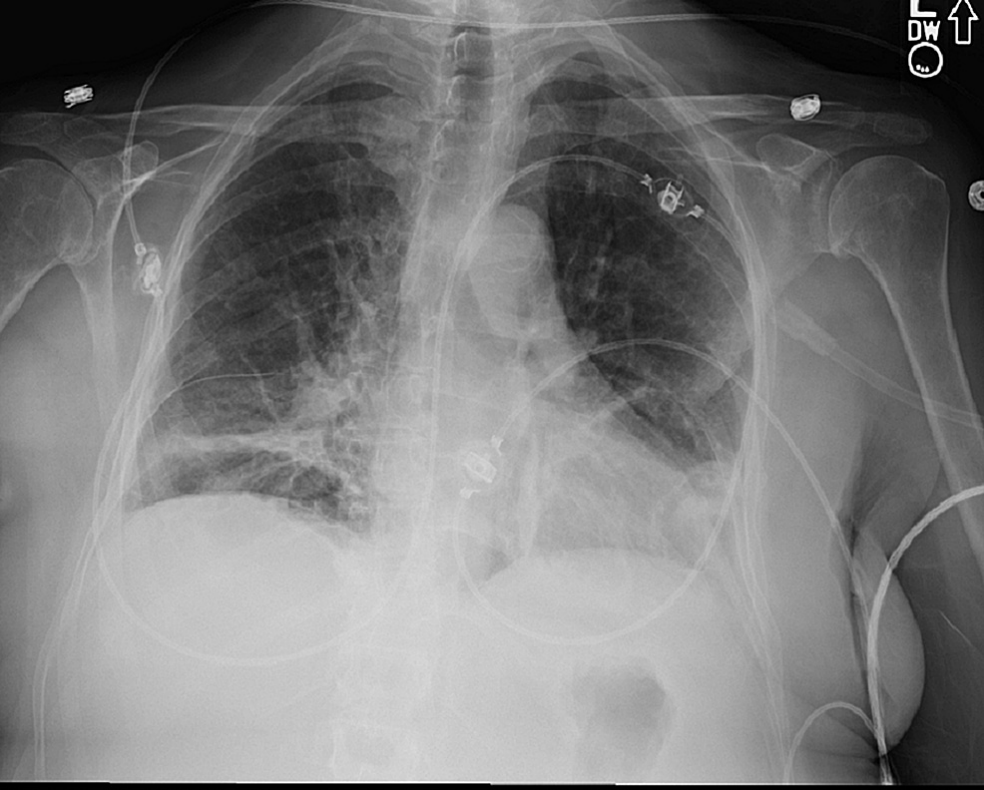
Chest radiograph on admission showing right perihilar and basilar airspace opacities concerning for pneumonia

Vitals were stable on presentation. Laboratory data was notable for mild leukopenia of 2.5 x 10^3^, platelets of 59 x 10^3^ (166 x 10^3^ two weeks prior), aspartate aminotransferase and alanine aminotransferase into the 100s, a total bilirubin of 1.8 mg/dL, and a gamma glutamyl transferase of 118 IU/L. Computed tomography (CT) of the head was negative for any acute intracranial process, and a chest x-ray revealed a right lower lobe opacification (Figure 1).

Over the next 24 hours the patient developed acute respiratory distress with fever, tachycardia, and hypotension refractory to fluid resuscitation. She was started on broad spectrum antibiotics with vasopressor support. Subsequent evaluation discovered *Klebsiella pneumoniae* in urine culture. At 72 hours, blood cultures remained normal, and the patient was weaned off vasopressor support. The patient additionally showed improvement in her mental status with treatment of her cystitis as well as overall progress in her clinical picture.

**Figure 2:**
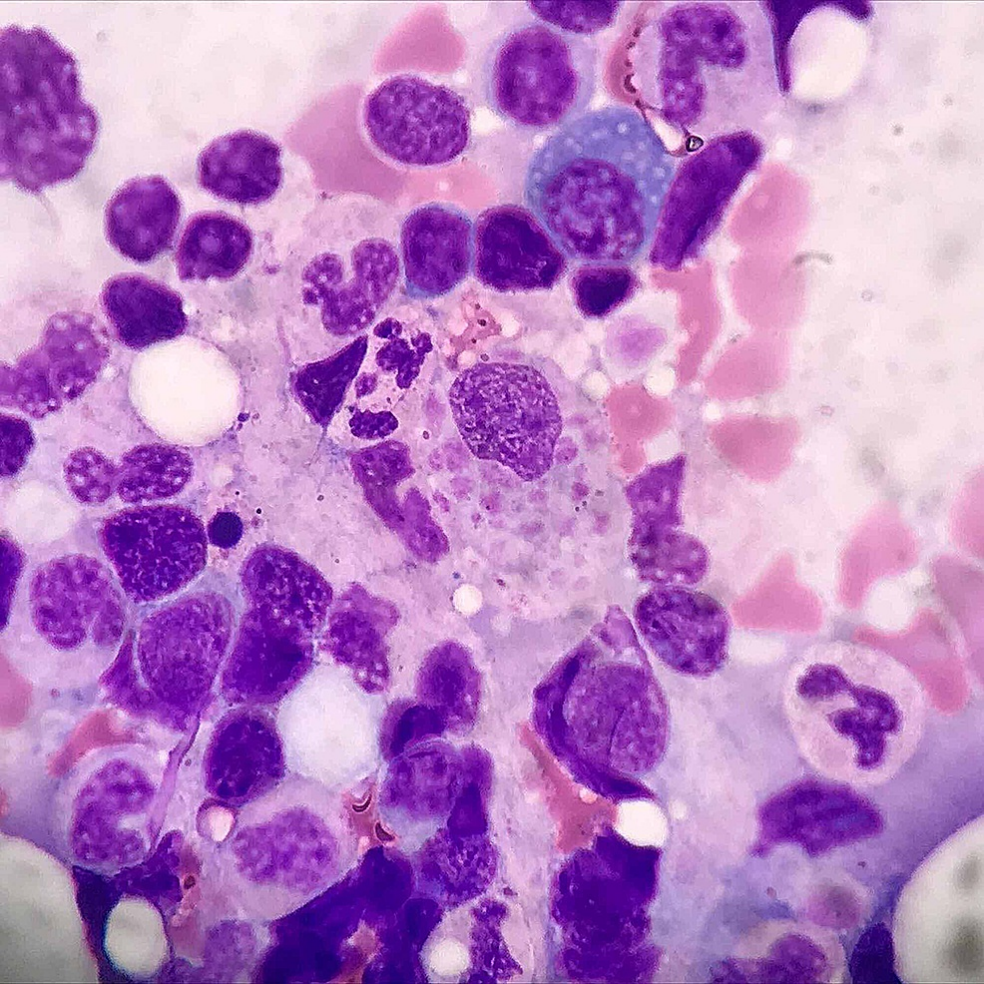
Bone marrow biopsy demonstrating trilineage dyspoiesis, hemophagocytosis of platelets, and nucleated red blood cells

**Figure 3:**
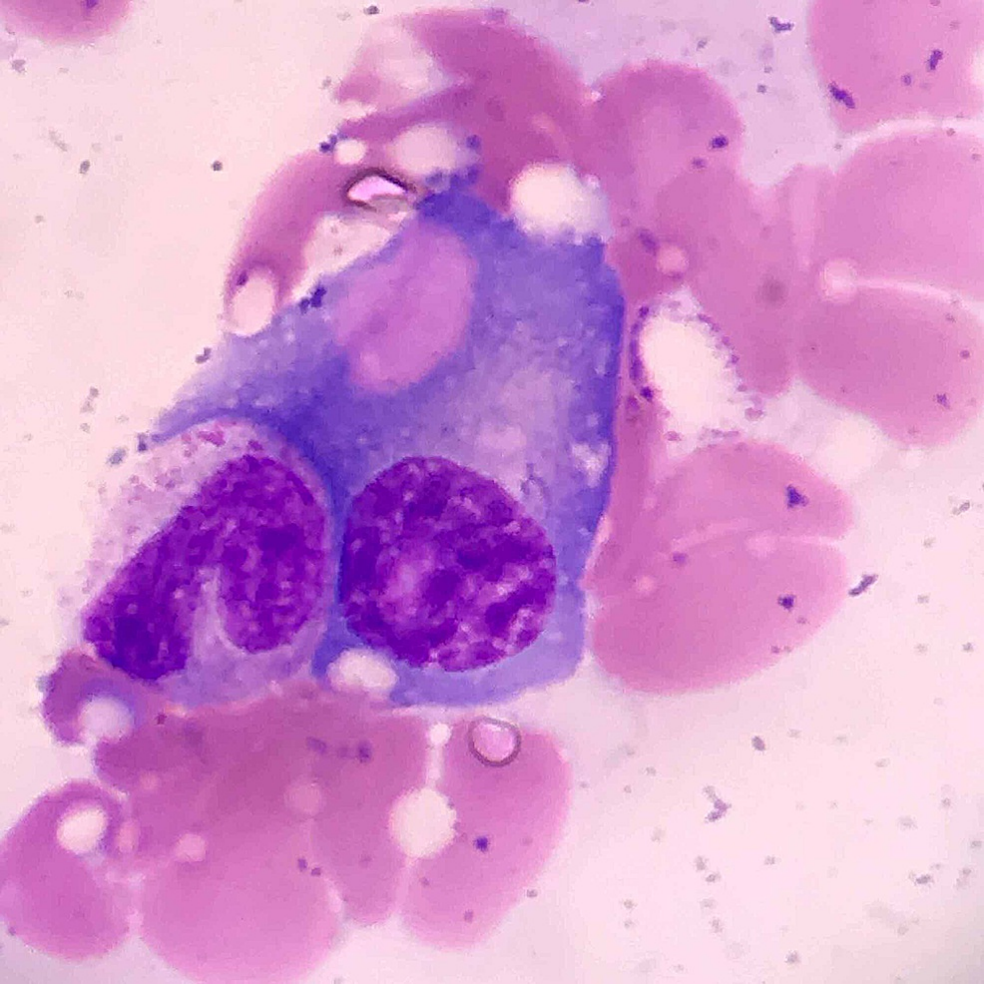
Bone marrow biopsy demonstrating plasma cell phagocytosis of a red blood cell

Despite the improvement, platelet count decreased to 16 x 10^3^ and hemoglobin from 13.9 g/dL to 7 g/dL without signs of active bleeding. Despite improvement in the patient’s presenting altered mental status after initiation of antibiotics, the cytopenias observed did not recover. No schistocytes nor spherocytes were on peripheral blood smear; fibrinogen was 830.0 mg/dL, and calculated 4T score was 2, with a normal lactate dehydrogenase (LDH), elevated haptoglobin, and absolute reticulocyte count of 0.2 (<2 indicating hypoproliferation). Ferritin was greater than 8,000 ng/m with an interleukin two receptor level of 4,490 U/mL (223-710 U/mL), fasting triglycerides of 275 mg/dL, and a negative platelet-factor-4 antibody. A bone marrow biopsy was performed showing frequent trilineage dyspoiesis, hemophagocytosis of platelets, and nucleated red blood cells (Figure 2) along with plasma cell hemophagocytosis (Figure 3). Flow cytometric analysis revealed no monoclonal cell population and no M-spike on serum protein electrophoresis, and her light chains were within normal limits, indicating no evident active myeloma disease.

The patient had a calculated HScore of 182 corresponding to a 70%-80% probability of hemophagocytic syndrome and met five out of eight criteria to make a definitive diagnosis of HLH: trilineage cytopenia, fasting triglycerides > 265mg/dL, hemophagocytosis in bone marrow, ferritin > 500ng/mL, and elevated soluble CD25 (IL-2 receptor).

Infectious etiologies for pancytopenia included respiratory and gastrointestinal virus panels in addition to laboratory assessments for hepatitis B and C, human simplex virus 1 and 2, cytomegalovirus, parvovirus, Epstein-Barr virus (EBV), COVID, Rocky Mountain Spotted Fever, typhus, leptospirosis, HIV, legionella pneumophilia, histoplasmosis, and *Streptococcus pneumonia*. All of the aforementioned infectious work-up was negative. Anti-nuclear antibody, double-stranded DNA, and rheumatoid factor testing returned negative. With no malignant or rheumatologic etiologies identified, the only remaining causative agent was immunomodulation secondary to lenalidomide.

A working diagnosis of lenalidomide-associated HLH with plasma cell hemophagocytosis was made. In addition to continued withholding lenalidomide, the patient started an eight-week dexamethasone taper with a 2.0 g/dL improvement in hemoglobin level and platelet increase to 58 x 10^3^ after one week. At eight weeks, her counts had normalized, and she had remained without laboratory evidence of MM recurrence.

## Discussion

HLH is not a single disease entity but a clinical syndrome defined by excessive activation of lymphocytes and macrophages resulting in elevated cytokine levels. HLH due to genetic etiologies is known as primary HLH. It can be divided into either familial HLH (HLH) or immune deficiencies such as Chédiak-Higashi syndrome (CHS), Griscelli syndrome (GS), or X-linked lymphoproliferative syndrome (XLP) [1,5].

The underlying pathophysiology of primary HLH is impaired NK cell function [6]. Hypersecretion of pro-inflammatory cytokines induces the hallmark inflammatory process of HLH [6]. Accumulation of the cytokines eventually leads cellular infiltrates into tissue, which can cause tissue necrosis and organ failure [6]. In primary HLH, NK cells fail to induce lysis of activated T-lymphocytes and histiocytes due to genetic mutations affecting perforin production and ultimately cytotoxic granule release [7]. Immune deficiencies linked with HLH, which are typically associated with pseudoalbinism, include Chédiak-Higashi syndrome (CHS), GS, and Hermansky-Pudlak syndrome type II [5,8,9]. The pathophysiology of HLH in CHS, GS, and Hermansky-Pudlak syndrome type II are due to insufficient and sometimes non-existent vesicle transport of cytotoxic granules, which further contributes to the pro-inflammatory milieu seen in HLH.

HLH-related diseases (secondary HLH) due to infectious, malignant, or autoimmune phenomena represent a significant subset of diseases and are the focus of this report. Secondary HLH encompasses a wide variety of underlying causes (infectious, autoimmune macrophage-associated hemophagocytic syndrome, malignancy-induced, immunomodulatory drug-induced) and has contributed to the development of novel T-cell engaging immunotherapies [1,2,4,6]. Infection-associated HLH has been documented mainly in case series with viral infections such as EBV, cytomegalovirus (CMV), and influenza [4]. Other common infectious etiologies include human immunodeficiency virus (HIV) [1,4]. Less common etiologies include *Mycobacterium tuberculosis*, rickettsia, leishmaniasis, and histoplasmosis [10]. EBV-associated HLH is most frequent in individuals with X-linked polydactyly (XPL) where there is a high risk for EBV reactivation due to impaired cytotoxic NK and T-cell function [10]. Macrophage activation syndrome is associated with autoimmune syndromes resulting in fatal pro-inflammatory condition thought to be a secondary defect in lymphocyte cytolytic activity [11]. Macrophage activation syndrome has been shown to propagate as a complication of systemic inflammatory disorders to include systemic juvenile idiopathic arthritis and systemic lupus erythematosus. Hemophagocytosis by macrophages is robustly associated with development of macrophage activation syndrome in patients with concomitant autoimmune diseases [12]. Much like HLH, in a subset of macrophage activation syndrome patients with altered cytolytic pathways, a pro-inflammatory cytokine environment ensues, which leads both to decreased activity of NK cells and inappropriate activation of macrophages contributing to multi-organ dysfunction [11-13].

Malignancy-associated HLH (Mal-HLH) remains the most common form of secondary HLH and has the worst prognosis of HLH subgroups [4]. Lymphomas remain the most frequent triggers of Mal-HLH based on a large series of studies [6]. Direct immune activation by transformed lymphocytes and/or loss of inhibitory immune function as a result of active malignancy remains a contributing factor to HLH activation [7,10]. Additionally, patients undergoing hematopoietic stem cell transplant are at a higher risk for HLH due to selective immune reconstitution [10].

In this patient, the leading pathophysiologic mechanism thought to be responsible for the propagation of secondary HLH was loss of immune hemostasis in the setting of previous hematopoietic stem cell transplant. The patient was also taking the immunomodulatory drug lenalidomide, which was thought to aggravate T-cell dysfunction and lower the threshold for triggering HLH. Limited data and case reports exist demonstrating causality between hematopoietic stem cell transplant and secondary HLH [14-16]. However, in the cases where it is seen, 12 days after receiving hematopoietic stem cells transplantation, a reactive hemophagocytic syndrome has developed thought to be secondary to high cytokine release by the peripheral blood stem cell graft [17]. A secondary HLH phenomenon developed after administration of dexamethasone, etoposide, and cyclosporine A to a patient with MM was believed to be more consistent with an immunomodulatory therapy-induced HLH [15]. It is thought that a hypersecretion of cytokines secondary to T-cell-activating immunotherapies may be inducing a cytokine release syndrome (CRS) causing an immunologic phenomenon similar to HLH. However, the description to lenalidomide being a causative agent for this phenomenon in the literature is limited.

Macrophage hemophagocytosis is commonly seen on bone marrow biopsy in patients with HLH [1,7]. Interestingly, the patient in this case displayed phagocytosis by plasma cells (Figure 3), which is a rare phenomenon that has been documented only a handful of times. When it has previously been seen, it was associated with active MM or a plasma cell dyscrasia [13,14,17]. In another case, HLH occurred after an autologous stem cell transplantation, which was believed to have caused an incomplete immune reconstitution and subsequently triggered plasma cell hemophagocytosis [16]. In these cases, active plasma cell leukemia or MM was present, which is contrary to our patient as she was without evidence of active disease. There is limited information known about this phenomenon, and it merits further evaluation and study.

## Conclusions

HLH is a syndrome of excessive inflammation and tissue destruction due to a state of dysregulated immune modulation. The hallmark of the condition of hemophagocytosis is red blood cells, platelets, and/or white blood cells creating a multiple lineage cytopenia. Etiologies of HLH are divided into primary and secondary; secondary causes typically occur in adulthood in the setting of a known trigger due to infection, malignancy, autoimmune dysfunction, or an immunodeficiency state. It is known from extensive case reviews that up to half of secondary HLH cases are due to underlying malignancy. In the patient described in this case, there was no evidence of active malignancy with the only offending agent being the immunomodulatory drug lenalidomide. It is notable that in her case, hemophagocytosis was seen by plasma cells without active MM, a phenomenon rarely seen in the current literature.
